# Students’ perspectives on undergraduate oral surgery education

**DOI:** 10.1186/s12909-019-1703-y

**Published:** 2019-07-18

**Authors:** Fatih Cabbar, Muammer Çağrı Burdurlu, Ceyda Ozcakir Tomruk, Begum Bank, Berkem Atalay

**Affiliations:** 1 Oral and Maxillofacial Surgery Department, Dentistry Faculty, University of Yeditepe, No:238 Bağdat Cd, Göztepe, 34728 Istanbul, Turkey; 2 Oral and Maxillofacial Surgery Department, Dentistry Faculty, University of Istanbul, Fatih, Istanbul, Turkey

**Keywords:** Dental students’ opinion, Dental students’ self-confidence, Instrument reliability, Oral surgery teaching

## Abstract

**Purpose:**

This study evaluated students’ perceptions of their self-confidence regarding aspects of their undergraduate oral and maxillofacial surgical training. It further aimed to develop a reliable Turkish version of the questionnaire originally developed by the Association of British Academic Oral Maxillofacial Surgeons (ABAOMS) Education Committee.

**Methods:**

A cross-sectional survey of 40 fourth-year and 47 fifth-year dentistry students of Yeditepe University Faculty of Dentistry in Turkey with a mean age of 23.30 ± 1.50 was conducted in January and February 2018. The ABAOMS questionnaire was adapted to the Turkish language and culture. The items were organized in five domains (general information, self-confidence in oral surgery, role of outreach, anatomy knowledge in relation to oral surgery, and career aspirations) with most response options on a five-point Likert-type scale. Reliability was assessed through an internal consistency analysis and a test-retest approach. Descriptive statistics, independent samples *t*-tests, and Chi-squared for contingency tests were used to examine the data.

**Results:**

Cronbach’s alpha coefficient on the questionnaire was 0.89. The responses reflected general agreement among the respondents. Females were significantly more self-confident than males. Although the fifth-year respondents were more self-confident than the fourth-year respondents on items regarding anatomy knowledge, fourth-year respondents were more self confident in forceps extractions. Other than that no major differences in self-confidence were found between the two groups.

**Conclusion:**

Though self-confidence was high regarding extraction of teeth and retained roots, the participants of this study lacked self-confidence in performing surgical extractions and its related procedures, recognition of malignancies, and ability to differentiate between pain of odontogenic or non-odontogenic origin. Female students were relatively more self-confident. Teaching should focus on practical applications that support students’ sense of self-confidence in their abilities. The Turkish version of the questionnaire was a reliable instrument.

## Background

Various dental curricula for undergraduate education in oral surgery recently have been developed and used. In addition, the published DentEd III-ADEE profile and competencies outline the major competencies to which all ADEE accredited institutions are expected to adhere, although achieving competencies might vary across schools [1]. A lively discourse on graduates’ abilities to perform oral surgery in private healthcare settings has accompanied these changes [2–5]. Curricula with clinical assessments allow educators to detect students who need additional support and identify highly proficient students for advancement to the next surgical challenge [6].

In addition to refined curricula and clinical assessments, questionnaires might be useful for identifying these students and determining the extent of their self-confidence regarding dental surgical procedures. Student feedback obtained via questionnaires also is important for monitoring educational quality because these data offer insights for enhancing teaching and course content effectiveness [7]. Some previous surveys have found a lack of self-confidence in the skills needed for surgical extractions [4, 8], and another study reported poor self-perceived understanding of oral surgery [9].

In Turkey, students’ perceptions of their oral surgery training have received little attention. There are no questionnaires originally in Turkish to assess students’ views on their oral surgery educations, and simply using a direct translation of existing questionnaires would be problematic because of language differences. However, the curriculum used by ADEE accredited schools in Turkey suggests that a Turkish-language version of the Association of British Academic Oral Maxillofacial Surgeons’ (ABAOMS) Education Committee questionnaire might be helpful for understanding Turkish students’ perceptions. The questionnaire comprises 17 questions in five domains designed to collect student’s self-‘perception of achieving core competencies in oral surgery training and included the year of study and gender data. It thoroughly covers students’ experiences in the core competencies outlined in the curriculum [9]. However, the reliability and reproducibility of a translated version is necessary.

This study’s main objective was to identify areas of strength and weakness among the fourth-year and fifth-year oral surgery students from their perspectives to establish a baseline for use in undergraduate oral surgery education. The second objective was to adapt the ABAOMS questionnaire regarding cultural characteristics to develop it into a valuable tool that dental schools in Turkey could use to evaluate aspects of their oral surgery education.

## Methods

This cross-sectional study was conducted at Yeditepe University Faculty of Dentistry in Turkey. Dental program is 5 years in Turkey, and oral surgery theoretical course starts in the fall semester of the third-year and continues together with the clinical course in the spring semester. The main core skills that are taught in third-year clinics are taking medical history, intra- and extra-oral investigations, diagnosis, forceps extraction and use of elevators. The third-year students are expected to perform at least 6 basic forceps extractions during the spring semester. At the following fourth-year and fifth-year, besides their further theoretical training, they are expected to perform at least 20 and 30 extractions including visible roots and third molars, respectively. Additionally, they perform minor surgical interventions such as suturing, mucosal flap reflection, under the supervision of experienced surgeons, during the all year. In the fifth-year, they obliged to visit the surgery room to observe different surgical procedures under both local and general anesthesia. They are allowed to observe consultant clinics throughout all years. Eventually, at the time of taking this survey, while the fourth-year participants had extracted about 16 teeth, the fifth-year participants had extracted about 41 teeth.

### Sampling procedure and participants

In January and February 2018, fourth-year and fifth-year undergraduate students at the dentistry school were recruited. Participants voluntarily consented to complete the questionnaire, filled it out, and were assured that their responses were anonymous. Age, grade, and gender data were obtained. Permission to use the questionnaire was electronically obtained from Dr. Macluskey [10]. The Ethical Committee at Yeditepe University approved the study in accordance with the Helsinki Declaration (Research no. 1369–761).

### Questionnaire adaptation process

Cultural adaptation of the questionnaire followed the process described by previous studies [11, 12]. First, three independent translators translated the questionnaire from English into Turkish. Then, the three translations were conformed to create the primary Turkish version. Disagreements were resolved and changes were made by discussing matters until consensus was reached. The face and content were evaluated by the researchers, all of whom are fluent in English and Turkish, which generated the second version. The second version was then back-translated by the same three independent translators, the translated versions were compared to the original questionnaire, and a semantic evaluation of every item in the Turkish version was performed. Last, the finalized Turkish questionnaire was pre-tested and reliability was assessed.

One of the independent translators was an experienced academician from the oral and maxillofacial surgery department with strong English competency (FC). The second expert was an academician in the same department who had lived in an English-speaking country for 3 years and earned a doctoral degree during that period (COT). The third expert was a professional translator. Disagreements were solved and changes made by discussing the subject in question until full agreement was reached during the second step, evaluation of face and content. Reliability was assessed through internal consistency and a reproducibility test-retest approach with a washout period of 1 month. After the first reliability evaluation, the students’ suggestions were reviewed and incorporated into the final version used for the study. No problems with the final version were found during the pre-test regarding comprehensibility or the completion process and it became the final Turkish version.

### Variables

The response options to the questionnaire items were on a Likert-type scale, except for three di/tri-chotomous questions. Altogether, there were 17 questions in five parts: (A) general information on the student’s gender and previous education, (B) confidence in oral surgical procedures, (C) role of outreach, (D) anatomy in relation to oral surgery teaching, and (E) career aspirations (Table 1).

**Table 1:** The original form and the Turkish translation of the questionnaire. Possible answers for each question were: a) Strongly agree; b) Agree; c) Neither agree or disagree; d) Disagree; e) Strongly disagree

B1. The teaching that I have received in oral surgery has given me sufficient knowledge to undertake independent practise. Oral cerrahi alanında almış olduğum eğitim, bağımsız uygulamaları üstlenmeme yetecek kadar bilgi sahibi olmamı sağlamıştır.	
B2. I feel confident that I could extract an upper single rooted tooth with an intact crown, in an otherwise intact dentition. Kuronu sağlam olan üst tek köklü dişi çekebilecek kadar kendime güveniyorum.	
B3. I feel confident that I could remove visible retained roots of an upper left first molar with elevators or forceps. Üst çene soldan ilk azı dişinin gözle görülür köklerini elevatör ve forseps yardımıyla çıkarabilecek kadar kendime güveniyorum	
B4. I feel confident to assess and perform the surgical management of a failed extraction (e.g. a lower second molar) necessitating: a) The raising of a mucoperiosteal flap Başarısız bir diş çekimini (örneğin alt çene ikinci azı dişi) değerlendirebilecek ve aşağıdakileri gerektiren cerrahi yönetimini uygulayacak kadar kendime güveniyorum: a) Mukoperiosteal flep kaldırılması	
B4b) Bone removal. Kemiğin kaldırılması	
B4c) Sectioning the tooth to facilitate elevation of the roots. Köklerin elevasyonunu kolaylaştırmak için dişin kesilmesi.	
B4d) Wound closure using appropriate suture materials. Uygun dikiş materyallerini kullanarak yaranın kapatılması	
B5. I feel confident to diagnose and manage acute pericoronitis. Akut perikoronit tanısını koyacak ve yönetimini üstlenecek kadar kendime güveniyorum.	
B6. I feel confident to manage haemorrhage from a socket. Diş çekim boşluğunda meydana gelen bir kanamayı yönetecek kadar kendime güveniyorum.	
B7. I feel confident to assess an impacted mandibular third molar with respect to guidelines and recognise the need for surgical removal. Gömülü mandibular üçüncü azı dişini doğru değerlendirecek ve cerrahi müdahale ile çıkarıp çıkarılmaması gerektiğine karar verecek kadar kendime güveniyorum.	
B8. I feel confident that I can recognize the clinical features of potentially malignant and malignant lesions of the oral cavity. Kötü huylu olma potansiyeli bulunan ve kötü huylu olan ağız boşluğu lezyonlarının klinik özelliklerini tanıyabilecek kadar kendime güveniyorum.	
B9. I feel confident that I can write an appropriate referral letter to a specialist in an appropriate time frame dependent on the clinical problem. Klinik soruna bağlı olarak uygun zaman diliminde bir uzmana sevk yazısı yazabilecek kadar kendime güveniyorum.	
B10. I feel competent to differentiate between pain of odontogenic and non-odontogenic origin. Odontojenik kaynaklı olan ve olmayan acıyı ayırt edebilecek kadar yeterli olduğumu düşünüyorum.	
C1. Where you involved in an outreach scheme? Bir sosyal yardım programında yer aldınız mı?	
C2. Did you carry out any extractions in outreach? Sosyal yardım programı kapsamında diş çektiniz mi?	
C3. Did you carry out any surgical extractions in outreach? Sosyal yardım programı kapsamında cerrahi müdahale ile diş çektiniz mi?	
D1. I believe my teaching in anatomy has been appropriate for my clinical needs in oral surgery. Anatomi alanındaki eğitimimin oral cerrahi alanındaki klinik ihtiyaçlarım için uygun olduğuna inanıyorum.	
D2. I am more confident about undertaking oral surgery because of my knowledge and understanding of head and neck anatomy. Kafa ve boyun anatomisi alanında bilgim dolayısıyla oral cerrahinin sorumluluğunu alma konusunda kendime daha çok güveniyorum.	
D3. The only anatomical knowledge needed for oral surgery is that of jaw and tooth morphology Oral cerrahi için gerekli olan tek anatomi bilgisi çene ve diş morfolojisidir.	
E1. Oral surgery is an enjoyable and rewarding discipline. Oral cerrahi eğlenceli ve tatmin edici bir disiplindir.	

### Methods of statistical analysis

The statistical analysis was performed using the Number Cruncher Statistical System (NCSS) 2007 Statistical Software (Utah, USA) program. Summary descriptive statistics (means and standard deviations), independent samples *t*-tests (to compare the two student groups), and Chi-squared for contingency tests (on categorical data) were performed. Pearson correlations were computed to assess the statistical relationships between the variables. Cronbach’s alpha was used to determine the questionnaire’s internal consistency, and alpha coefficients greater than 0.7 were acceptable [13]. Spearman’s rank-order correlation, intra-class correlation coefficients, and a 95% confidence interval were used to evaluate test-retest reliability. Data on the questions with response options of “yes” or “no” were assessed using a weighted kappa test κw. The statistical significance cutoff level was 0.05.

## Results

Table 2 presents the results on test-retest reliability, intra-class correlation, and the weighted kappa coefficient. The Cronbach’s alpha was 0.89.

**Table 2:** The test-retest reliability, intra-class correlation, and weighted kappa coefficient of the questionnaire

	Intra-class correlation coefficient	95%	Spearman correlation
B1. The teaching that I have received in oral surgery has given me sufficient knowledge to undertake independent practise.	0.962	(0.942–0.975)	0.940
B2. I feel confident that I could extract an upper single rooted tooth with an intact crown, in an otherwise intact dentition.	0.896	(0.841–0.932)	0.812
B3. I feel confident that I could remove visible retained roots of an upper left first molar with elevators or forceps.	0.952	(0.926–0.968)	0.914
B4. I feel confident to assess and perform the surgical management of a failed extraction (e.g. a lower second molar) necessitating: a) The raising of a mucoperiosteal flap	0.989	(0.983–0.993)	0.978
B4b) Bone removal.	0.986	(0.979–0.991)	0.972
B4c) Sectioning the tooth to facilitate elevation of the roots.	0.980	(0.97–0.987)	0.961
B4d) Wound closure using appropriate suture materials.	0.976	(0.963–0.984)	0.953
B5. I feel confident to diagnose and manage acute pericoronitis.	0.978	(0.967–0.986)	0.958
B6. I feel confident to manage haemorrhage from a socket.	0.978	(0.967–0.986)	0.958
B7. I feel confident to assess an impacted mandibular third molar with respect to guidelines and recognise the need for surgical removal.	0.976	(0.964–0.984)	0.954
B8. I feel confident that I can recognise the clinical features of potentially malignant and malignant lesions of the oral cavity.	0.976	(0.964–0.985)	0.954
B9. I feel confident that I can write an appropriate referral letter to a specialist in an appropriate time frame dependent on the clinical problem.	0.948	(0.920–0.966)	0.901
B10. I feel competent to differentiate between pain of odontogenic and non-odontogenic origin.	0.989	(0.983–0.993)	0.980
D1. I believe my teaching in anatomy has been appropriate for my clinical needs in oral surgery.	1.000	(1.000–1.000)	1.000
D2. I am more confident about undertaking oral surgery because of my knowledge and understanding of head and neck anatomy.	1.000	(1.000–1.000)	1.000
D3. The only anatomical knowledge needed for oral surgery is that of jaw and tooth morphology	1.000	(1.000–1.000)	1.000
E1. Oral surgery is an enjoyable and rewarding discipline.	1.000	(1.000–1.000)	1.000
C1. Where you involved in an outreach scheme?	κ_w_:1.000		
C2. Did you carry out any extractions in outreach?	κ_w_:1.000		
C3. Did you carry out any surgical extractions in outreach?	κ_w_:1.000		

### Section a: general information

Table 3 presents the distributions of variables used in the study. There were 102 students in total. Of these, 55 were fourth-year and 47 were fifth-year. Of the 55 fourth-year and 47 fifth-year students, 15 fourth-year students did not return completed questionnaires, resulting a response rate of 85.3% and final sample size of 87. The sample comprised 25 male (28.7%) and 62 female (71.3%) respondents with a mean age of 23.30 ± 1.5. Of the respondents, 40 (46%) were fourth-year and 47 (54%) were fifth-year students. Eleven male (27.5%) and 29 female (72.5%) respondents were fourth-year students; there were 14 male (29.8%) and 33 female (70.2%) fifth-year students. There was no statistically significant gender difference by school year (*p* = 0.874).

**Table 3:** The frequency of answers for each of the questions

	Strongly agree	Agree	Neither agree or disagree	Disagree	Strongly disagree	Mean ± SD	Median	Min.	Max.
B1. The teaching that I have received in oral surgery has given me sufficient knowledge to undertake independent practise.	12 (13.79%)	45 (51.72%)	13 (14.94%)	17 (19.54%)	–	2.4 ± 0.96	2	1	4
B2. I feel confident that I could extract an upper single rooted tooth with an intact crown, in an otherwise intact dentition.	40 (45.98%)	41 (47.13%)	5 (5.75%)	–	1 (1.15%)	1.63 ± 0.7	2	1	5
B3. I feel confident that I could remove visible retained roots of an upper left first molar with elevators or forceps.	13 (14.94%)	34 (39.08%)	21 (24.14%)	16 (18.39%)	3 (3.45%)	2.56 ± 1.06	2	1	5
B4. I feel confident to assess and perform the surgical management of a failed extraction (e.g. a lower second molar) necessitating: a) The raising of a mucoperiosteal flap	8 (9.2%)	18 (20.69%)	24 (27.59%)	11 (12.64%)	26 (29.89%)	3.33 ± 1.34	3	1	5
B4b) Bone removal.	5 (5.75%)	16 (18.39%)	20 (22.99%)	9 (10.34%)	37 (42.53%)	3.66 ± 1.35	4	1	5
B4c) Sectioning the tooth to facilitate elevation of the roots.	5 (5.75%)	21 (24.14%)	22 (25.29%)	12 (13.79%)	27 (31.03%)	3.4 ± 1.31	3	1	5
B4d) Wound closure using appropriate suture materials.	16 (18.39%)	40 (45.98%)	17 (19.54%)	6 (6.9%)	8 (9.2%)	2.43 ± 1.15	2	1	5
B5. I feel confident to diagnose and manage acute pericoronitis.	10 (11.49%)	45 (51.72%)	20 (22.99%)	11 (12.64%)	1 (1.15%)	2.4 ± 0.9	2	1	5
B6. I feel confident to manage haemorrhage from a socket.	14 (16.09%)	45 (51.72%)	19 (21.84%)	8 (9.2%)	1 (1.15%)	2.28 ± 0.89	2	1	5
B7. I feel confident to assess an impacted mandibular third molar with respect to guidelines and recognise the need for surgical removal.	19 (21.84%)	40 (45.98%)	14 (16.09%)	10 (11.49%)	4 (4.6%)	2.31 ± 1.08	2	1	5
B8. I feel confident that I can recognise the clinical features of potentially malignant and malignant lesions of the oral cavity.	10 (11.49%)	33 (37.93%)	23 (26.44%)	11 (12.64%)	10 (11.49%)	2.75 ± 1.17	3	1	5
B9. I feel confident that I can write an appropriate referral letter to a specialist in an appropriate time frame dependent on the clinical problem.	16 (18.39%)	48 (55.17%)	18 (20.69%)	3 (3.45%)	2 (2.3%)	2.16 ± 0.85	2	1	5
B10. I feel competent to differentiate between pain of odontogenic and non-odontogenic origin.	7 (8.05%)	33 (37.93%)	29 (33.33%)	11 (12.64%)	7 (8.05%)	2.75 ± 1.05	3	1	5
D1. I believe my teaching in anatomy has been appropriate for my clinical needs in oral surgery.	11 (12.64%)	42 (48.28%)	18 (20.69%)	5 (5.75%)	11 (12.64%)	2.57 ± 1.18	2	1	5
D2. I am more confident about undertaking oral surgery because of my knowledge and understanding of head and neck anatomy.	11 (12.64%)	26 (29.89%)	25 (28.74%)	6 (6.9%)	19 (21.84%)	2.95 ± 1.33	3	1	5
D3. The only anatomical knowledge needed for oral surgery is that of jaw and tooth morphology	5 (5.75%)	6 (6.9%)	6 (6.9%)	23 (26.44%)	47 (54.02%)	4.16 ± 1.18	5	1	5
E1. Oral surgery is an enjoyable and rewarding discipline.	28 (32.18%)	40 (45.98%)	11 (12.64%)	1 (1.15%)	7 (8.05%)	2.07 ± 1.11	2	1	5
Is the questionnaire clearly understandable?	64 (94.12%)	4 (5.88%)							

### Section B: confidence in oral surgical procedures

The majority of the respondents reported self-confidence in performing extractions of upper teeth with single roots and visible roots of an upper molar, with elevators or forceps (Question B2 = 93.1% and Question B3 = 54%), although they were more confident regarding teeth with single roots. Large proportions of the respondents either totally or strongly disagreed (lacked self-confidence) regarding surgical skills related to performing procedures related to surgical extractions (Question B4a: mucosal flap reflection, Question B4b: bone removal, and Question B4c: root sectioning), but about two-thirds of the respondents (64.4%) reported a sense of self-confidence in the ability to suture the wounds (Question B4d).

About two-thirds of the respondents reported a sense of self-confidence regarding diagnosing and managing pericoronitis (63.2%), managing haemorrhage (67.8%), and assessing impacted third molars (67.8%). In addition, about one-half (49.4%) felt confident in their ability to recognize malignant lesions, about three-quarters (73.6%) believed that they could write a referral to a specialist, and 46% felt self-confident about their ability to differentiate between pain of an odontogenic from that of a non-odontogenic origin. Questions B4a (mucosal flap reflection), B8 (recognition of malignancies), B10 (differentiate between pain of odontogenic and non-odontogenic origin), and D2 (anatomical knowledge for performing surgery) received the most “neither agree nor disagree” responses.

### Section C: role of outreach in oral surgery teaching

There was no outreach program for oral and maxillofacial surgery training.

### Anatomy teaching in relation to oral surgery

About 60.9% of the respondents reported that their anatomy courses had been adequate. However, just 42.5% felt confident in what they had learned. In response to the question asking whether only tooth-related and jaw-related anatomical knowledge was sufficient knowledge for performing oral surgery, 80.5% of the respondents disagreed.

### Section E: career aspirations

The majority (78.2%) of the students responded that oral surgery is an enjoyable and rewarding discipline.

### Correlations

Statistically significant correlations were found between all aspects of self-confidence in surgical skills (Question B4a: mucosal flap reflection, Question B4b: bone removal, Question B4c: root sectioning, and Question B4d: suture the wounds) and self-confidence in all aspects of forceps extractions (Question B2: extraction of tooth with single root and Question B3: extraction of visible root), except regarding Question B2, which only correlated with Question B3. The associations between anatomy knowledge in relation to oral surgery (Question D1: appropriate anatomical teaching for clinical needs, Question D2: performing oral surgery based on anatomical knowledge, and Question D3: the only knowledge necessary for oral surgery relates to jaw and tooth anatomy) and self-confidence in surgical skills also were statistically significant, except the relation between Question B4d and Question D3.

Statistically significant gender differences were found regarding most of the questions in Sections B and D, and females generally responded more positively than males (Table 4). Most of the items were not significantly different regarding the respondents’ school year, but fourth-year respondents were more confident regarding extraction of tooth with single root (Questions B2), determining the need to extract impacted third molar (Question B7), and performing oral surgery based on anatomical knowledge (Question D2), whereas fifth-year respondents were more confident regarding Question D3 (the only knowledge necessary for oral surgery relates to jaw and tooth anatomy).

**Table 4:** The differences between responds of female and male students

	Female n: 62	Male n: 25	*p*
B1. The teaching that I have received in oral surgery has given me sufficient knowledge to undertake independent practise.	2.60 ± 0.97	1.92 ± 0.76	**0.002**
B2. I feel confident that I could extract an upper single rooted tooth with an intact crown, in an otherwise intact dentition.	1.71 ± 0.76	1.44 ± 0.51	0.105
B3. I feel confident that I could remove visible retained roots of an upper left first molar with elevators or forceps.	2.74 ± 1.01	2.12 ± 1.09	**0.013**
B4. I feel confident to assess and perform the surgical management of a failed extraction (e.g. a lower second molar) necessitating: a) The raising of a mucoperiosteal flap	3.50 ± 1.3	2.92 ± 1.38	0.068
B4b) Bone removal.	3.85 ± 1.2	3.16 ± 1.57	**0.028**
B4c) Sectioning the tooth to facilitate elevation of the roots.	3.60 ± 1.18	2.92 ± 1.5	**0.028**
B4d) Wound closure using appropriate suture materials.	2.63 ± 1.19	1.92 ± 0.86	**0.008**
B5. I feel confident to diagnose and manage acute pericoronitis.	2.52 ± 0.9	2.12 ± 0.83	0.061
B6. I feel confident to manage haemorrhage from a socket.	2.40 ± 0.91	1.96 ± 0.74	**0.034**
B7. I feel confident to assess an impacted mandibular third molar with respect to guidelines and recognise the need for surgical removal.	2.50 ± 1.14	1.84 ± 0.75	**0.009**
B8. I feel confident that I can recognise the clinical features of potentially malignant and malignant lesions of the oral cavity.	2.90 ± 1.17	2.36 ± 1.11	**0.049**
B9. I feel confident that I can write an appropriate referral letter to a specialist in an appropriate time frame dependent on the clinical problem.	2.15 ± 0.81	2.20 ± 0.96	0.787
B10. I feel competent to differentiate between pain of odontogenic and non-odontogenic origin.	2.92 ± 1.06	2.32 ± 0.9	**0.015**
D1. I believe my teaching in anatomy has been appropriate for my clinical needs in oral surgery.	2.76 ± 1.17	2.12 ± 1.09	**0.021**
D2. I am more confident about undertaking oral surgery because of my knowledge and understanding of head and neck anatomy.	3.13 ± 1.29	2.52 ± 1.36	0.052
D3. The only anatomical knowledge needed for oral surgery is that of jaw and tooth morphology	4.29 ± 0.98	3.84 ± 1.55	0.108
E1. Oral surgery is an enjoyable and rewarding discipline.	2.16 ± 1.18	1.84 ± 0.9	0.223

Independed t-test

Data in bold are significant *p* values

## Discussion

Students were self-confident about their abilities to extract teeth and retained roots, however they lacked self-confidence in performing surgical extractions and its related procedures.

This study’s findings were similar to findings of Al-Dajani M., Wanigasooriya N., and Macluskey et al., which found that the majority of the participants felt confident performing forceps extractions and that their oral surgery education had given them sufficient self-confidence [9, 14, 15]. A significant positive correlation was found between the students’ self-confidence in performing forceps extractions and performing surgical extraction, but self-confidence about surgical extractions was not as strong as it was for forceps extractions. In line with this finding, Al-Dajani M. reported relatively less confidence in surgical extractions or root sectioning than in simple extractions [14]. This relatively low self-confidence in the ability to perform surgical extractions was highlighted by several previous studies [4, 8, 9]. Durham et al. reported that students lacked self-confidence regarding this procedure because surgical extraction is one of the most invasive procedures in clinical settings, and they are intimidated by it even when they are competent [16]. On the other hand, this finding might reflect a lack of practical experience in surgical extraction, because findings of this study showed that self-confidence regarding surgical extractions significantly correlated with self-confidence regarding mucosal flap reflection, bone removal, sectioning the roots, and suturing. Moreover, the lowest levels of self-confidence were reported about mucosal flap reflection, bone removal, and sectioning the roots. This might result from the lack of suitable cases for undergraduates to participate in [16, 17]. However, this could also possibly be due to postgraduate oral surgery students, who may be performing these extractions instead of undergraduates as part of their education program. Unfortunately, it is not always possible to quantify these surgical interventions. Therefore, recording these surgical interventions in an individual’s logbook separately could be useful for further studies [18].

The majority of the respondents reported confidence in haemorrhage control, diagnosing acute pericoronitis, making decisions to extract impacted third molars, and making referrals to specialists, which was a reassuring result. A previous study found low levels of self-confidence regarding the ability to recognize potentially malignant lesions and the ability to differentiate between odontogenic and non-odontogenic pain [9], which our findings supported. In another study, the lowest confidence level was observed in biopsy procedures, which was also a topic related with the oral lesions [14]. In line with those studies, Wanigasooriya N. reported low confidence of students in managing oral mucosal lesions, recognizing pre-cancerous conditions in the mouth and diagnosing oro-facial pain of dental origin [15]. It is possible that these findings reflect the respondents’ lack of exposure to these lesions at this point in their studies, but it might imply that a targeted approach to training would help students achieve a balanced sense of self-confidence [9].

One important factor for developing self-confidence is sufficient knowledge and understanding of basic medical science, of which anatomical knowledge is an important component [9]. To the best of our knowledge, ours is the first study to evaluate dental students’ perceptions of their anatomical knowledge, in Turkey. The majority of the respondents felt self-confident that their anatomical education was appropriate to their future practice. However, few of them felt confident undertaking oral surgery based on their current knowledge of head and neck anatomy. Therefore, this aspect of the curriculum should be addressed. Anatomy usually is taught during the first and second years. In addition to the basic training, anatomy is vertically integrated during later stages in the oral surgery lessons. However, apparently, more focus in this area would be useful to refresh previously learned information. Thomas et al. suggested that anatomy based knowledge should be continuously refreshed amongst dentists because of the increasing numbers of hospitalized cases arising from dental abscess [19]. Along these lines, one important result was that the majority of the respondents disagreed that the only knowledge necessary for oral surgery relates to jaw and tooth anatomy, which was supported by our finding of a positive correlation between self-confidence in anatomical knowledge and in surgical skills (such as mucosal flap reflection and bone removal, root sectioning, or suturing).

Female students in our study reported more self-confidence than the males regarding all aspects of extraction, and most aspects of the whole questionnaire. This may be the result of female students also being more confident in anatomical aspects. In addition, it is possible that female students are more successful in theoretical lessons. However, the design of this study limits our ability to make such conclusions.

One might assume that upper-level students might have relatively more self-confidence than their lower-level counterparts because they have had relatively more clinical experience. Al-Dajani N. suggested that the more teeth the students extracted, the more confidence they achieved [14]. However, we found that the fourth-year respondents were more self-confident than the fifth-year respondents regarding performing forceps extractions and determining the need to extract impacted third molars. Fifth-year respondents were more self-confident in undertaking oral surgery with regard to their knowledge of anatomy and more of them disagreed that head and neck anatomy knowledge is enough for oral surgery. These findings might reflect their greater practical experience because they likely had performed more extractions and, therefore, faced more complications, which might give them more prudence in extractions and greater clinical-oriented anatomy knowledge.

This study’s second objective was to develop a questionnaire in the Turkish language that could be used by dental schools to evaluate aspects of their oral surgery educational outcomes. Overall, the study’s questionnaire had internal consistency to evaluate perceptions of oral surgery education and self-confidence.

One of this study’s limitations is that the questionnaire was anonymous, which makes it impossible to characterize the abilities of the non-respondents in areas of the oral surgery or self-confidence. Additionally, self-reporting on self-confidence means that the respondents did not necessarily demonstrate clinical competence that matched their self-reported claims. Therefore, these results should be interpreted with caution. This is the first study in Turkey to evaluate dental students’ perceptions of their oral surgery training. Therefore, it is a benchmark study providing a reference for future similar studies. However, because the sample was from one dental program and the sample was relatively small, generalizability is problematic. Because this study confirmed the reliability of the questionnaire, we plan to conduct a national survey and to continue working on this line of research. Further, the authors have been developing a standardized rotational community outreach program.

## Conclusions

Female students were more confident than male students in most areas, such as undertaking independent practice, visible root removal, bone removal, root sectioning, wound suturing, managing haemorrhage, assessing an impacted mandibular third molar, recognizing malignancies, and differentiation of pain of odontogenic and non-odontogenic origin. There is a need to improve training in the areas of surgical extractions and procedures related to surgical extraction, viz. raising a mucoperiosteal flap, bone removal, root sectioning, wound suturing; recognizing malignancies, and differentiation of pain of odontogenic and non-odontogenic origin. To address the problems revealed by this study, students have been assigned to oral pathology internships, and case-based lectures. The employment of this version of the questionnaire as a measurement for oral surgery curricula and students’ self-confidence is feasible, as long as the limitations are taken into consideration.

## Data Availability

The data used in this study are not publicly available for privacy reasons, but data can be made available from the corresponding author on request.

## Abbreviations

ABAOMS: Association of British Academic Oral Maxillofacial Surgeons
ADEE: Association for Dental Education Europe
NCSS: Number Cruncher Statistical System.
